# Palmitoleate Induces Hepatic Steatosis but Suppresses Liver Inflammatory Response in Mice

**DOI:** 10.1371/journal.pone.0039286

**Published:** 2012-06-29

**Authors:** Xin Guo, Honggui Li, Hang Xu, Vera Halim, Weiyu Zhang, Huan Wang, Kuok Teong Ong, Shih-Lung Woo, Rosemary L. Walzem, Douglas G. Mashek, Hui Dong, Fuer Lu, Lai Wei, Yuqing Huo, Chaodong Wu

**Affiliations:** 1 Intercollegiate Faculty of Nutrition, Department of Nutrition and Food Science, Texas A&M University, College Station, Texas, United States of America; 2 Department of Medicine, the University of Minnesota, Minneapolis, Minnesota, United States of America; 3 Department of Food Science and Nutrition, the University of Minnesota, St. Paul, Minnesota, United States of America; 4 Institute of Integrated Chinese and Western Medicine, Tongji Hospital, Huazhong University of Science and Technology Tongji Medical College, Wuhan, China; 5 Institute of Hepatology, Peking University Health Science Center, Beijing, China; 6 Department of Cellular Biology and Anatomy, Georgia Health Sciences University, Augusta, Georgia, United States of America; Pennington Biomedical Research Center, United States of America

## Abstract

The interaction between fat deposition and inflammation during obesity contributes to the development of non-alcoholic fatty liver disease (NAFLD). The present study examined the effects of palmitoleate, a monounsaturated fatty acid (16∶1n7), on liver metabolic and inflammatory responses, and investigated the mechanisms by which palmitoleate increases hepatocyte fatty acid synthase (FAS) expression. Male wild-type C57BL/6J mice were supplemented with palmitoleate and subjected to the assays to analyze hepatic steatosis and liver inflammatory response. Additionally, mouse primary hepatocytes were treated with palmitoleate and used to analyze fat deposition, the inflammatory response, and sterol regulatory element-binding protein 1c (SREBP1c) activation. Compared with controls, palmitoleate supplementation increased the circulating levels of palmitoleate and improved systemic insulin sensitivity. Locally, hepatic fat deposition and SREBP1c and FAS expression were significantly increased in palmitoleate-supplemented mice. These pro-lipogenic events were accompanied by improvement of liver insulin signaling. In addition, palmitoleate supplementation reduced the numbers of macrophages/Kupffer cells in livers of the treated mice. Consistently, supplementation of palmitoleate decreased the phosphorylation of nuclear factor kappa B (NF-κB, p65) and the expression of proinflammatory cytokines. These results were recapitulated in primary mouse hepatocytes. In terms of regulating FAS expression, treatment of palmitoleate increased the transcription activity of SREBP1c and enhanced the binding of SREBP1c to FAS promoter. Palmitoleate also decreased the phosphorylation of NF-κB p65 and the expression of proinflammatory cytokines in cultured macrophages. Together, these results suggest that palmitoleate acts through dissociating liver inflammatory response from hepatic steatosis to play a unique role in NAFLD.

## Introduction

Obesity greatly increases the incidence of non-alcoholic fatty liver disease (NAFLD), which is defined by fat deposition in hepatocytes (hepatic steatosis) [1], [2]. When the liver develops overt inflammation and damage, simple steatosis progresses to steatohepatitis, which is increasingly recognized as an essential causal factor of terminal liver diseases such as cirrhosis and hepatocellular carcinoma [2], [3]. A number of approaches including weight loss, metformin, insulin sensitization by thiazolidinediones, bariatric surgery, and liver transplantation have been considered for managing NAFLD [3]–[7]. However, the effective treatment for steatohepatitis is still lacking due largely to the absence of a clear understanding of how simple steatosis progresses to steatohepatitis.

Because NAFLD is highly prevalent in obese populations [6], insulin resistance, a common consequence of obesity, is thought to critically contribute to the pathogenesis of NAFLD. Mechanistically, insulin resistance at both hepatic and systemic levels, along with hyperinsulinemia, acts to increase the expression of genes for lipogenic enzymes such as acetyl-CoA carboxylase 1 (ACC1) and fatty acid synthase (FAS) [8], [9] and to decrease the expression of genes for enzymes of fatty acid oxidation including carnitine palmitoyltransferase 1a (CPT1a) [10]. These changes, in turn, bring about hepatic steatosis. As a primary “hit”, fat deposition is sufficient to trigger inflammatory responses as evidenced in cultured hepatocytes [11], [12], and to induce liver insulin resistance by activating protein kinase Cε in mice [13]. Moreover, fat deposition makes hepatocytes more vulnerable to a “second hit”, i.e., inflammatory mediators derived from adipose tissue during obesity [2]. When combined, these two “hits” exacerbate aspects of NAFLD, in particular liver inflammatory response, and advance steatosis to steatohepatitis. As reviewed elsewhere, there likely exists a vicious cycle among hepatocyte fat deposition, liver inflammatory response, and hepatic insulin resistance in the development of steatohepatitis [1], [2]. At this point, clear-cut causal relationships among aspects of NAFLD within the vicious cycle remain to be investigated.

Palmitoleate is a monounsaturated fatty acid (16∶1n7) that is available from dietary sources and is also produced endogenously by adipocytes. Since the characterization of palmitoleate as a bioactive lipid that coordinates metabolic crosstalk between the liver and adipose tissue [14], increased attention has paid to the pathophysiological relevance of palmitoleate to a wide variety of metabolic diseases including NAFLD. However, subsequent studies provide controversial findings regarding the effects of palmitoleate on hepatic steatosis and/or lipogenesis that critically contribute to the development of NAFLD. As documented in two lines of mouse studies [14], [15], palmitoleate inhibits hepatic FAS expression, and is thought to decrease hepatic steatosis. In contrast, human studies demonstrate that the circulating levels of palmitoleate positively correlate with the degree of hepatic steatosis [16], as well as adiposity that promotes fat deposition in hepatocytes [17], [18]. A direct effect of palmitoleate on inducing hepatocyte fat deposition has also been documented [19], but lacks *in vivo* validation. To date, precisely how palmitoleate regulates hepatic FAS expression in relation to lipogenesis is not clear. Palmitoleate is also shown to decrease palmitate-induced phosphorylation of c-Jun N-terminal kinase (JNK) in Huh7 hepatocytes [19]. This finding is exciting; given the essential role for inflammation in the development of steatohepatitis. However, the anti-inflammatory effect of palmitoleate has not yet been validated *in vivo*. The present study provides evidence to support a unique role for palmitoleate in dissociating liver inflammatory response from hepatic steatosis. Additionally, a novel mechanism for palmitoleate induction of FAS expression is investigated.

## Results

### Palmitoleate Supplementation Improves Systemic Insulin Sensitivity in LFD-fed Mice

**Figure 1:**
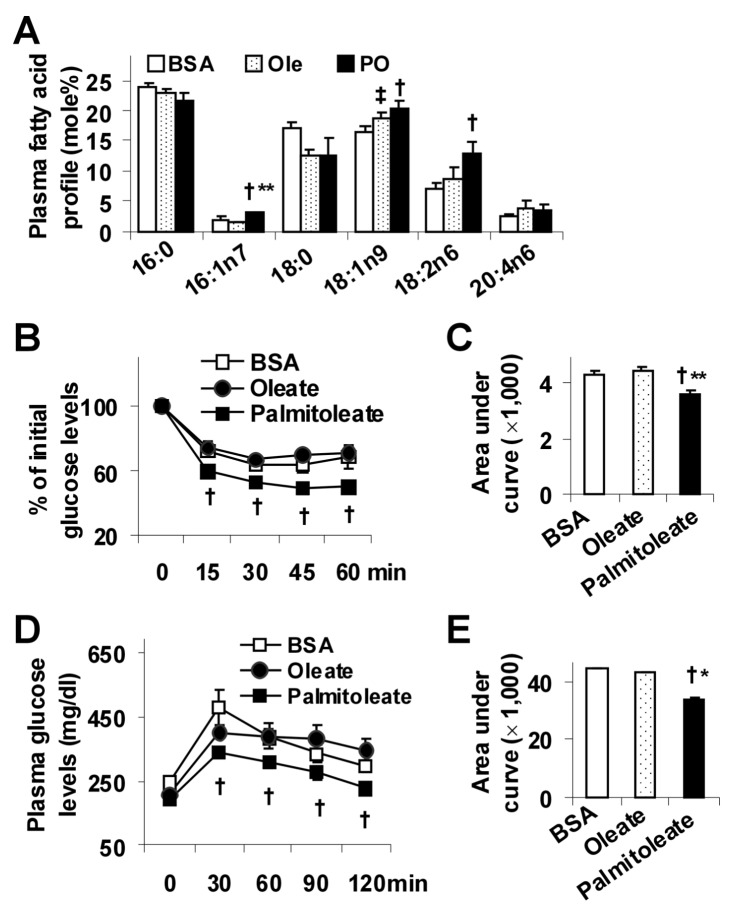
Improvement of systemic insulin sensitivity in LFD-fed mice. Male C57BL/6J mice, at 5–6 weeks of age, were fed a low-fat diet for 12 weeks and supplemented with palmitoleate (PO), oleate (Ole), or bovine serum albumin (BSA) for the last 4 weeks. (A) Plasma lipid profile. (B) Insulin tolerance tests (ITT). (C) Area under curve (AUC) was calculated based on ITT. (D) Glucose tolerance tests (GTT). (E) AUC was calculated based on GTT. For B and D, mice were fasted for 4 hrs and received an intraperitoneal injection of insulin (0.5 U/kg) (B) or glucose (2 g/kg) (D). Data are means ± SE, n = 4–6. ^†^, *P*<0.05 palmitoleate vs. BSA (in A – E) or oleate for the same time point (in B and D); *, *P*<0.05 and **, *P*<0.01 palmitoleate vs. oleate (in A, C, and E); ^‡^, *P*<0.05 oleate vs. BSA (in A).

Wild-type mice were fed a low-fat diet (LFD, Table 1) and treated with or without palmitoleate. Since palmitoleate was conjugated with bovine serum albumin (BSA), the latter was used as negative control. Additionally, oleate was used as fatty acid control. Compared with controls, palmitoleate supplementation significantly increased plasma levels of palmitoleate (Figure 1A) without significantly altering liver lipid profile (Figure S1). Consequently, palmitoleate-supplemented mice, but not control mice, showed improvement in systemic insulin sensitivity and glucose metabolic homeostasis (Figure 1B–E, and Table 2). Because food intake of palmitoleate-supplemented mice did not differ from that of control mice, the observed insulin-sensitizing effect, as well as alterations of hepatic steatosis and liver inflammatory response upon palmitoleate supplementation (see below), is not due to a decrease in energy intake.

**Table 1:** Composition of diets.*

	*LFD*		*HFD*	
	g%	kcal%	g%	kcal%
Casein	18.96	19.72	25.84	19.72
L-Cystine	0.28	0.30	0.39	0.30
Corn Starch	29.86	31.06	0.00	0.00
Maltodextrin	3.32	3.45	16.15	12.32
Sucrose	33.17	34.51	8.89	6.78
Cellulose	4.74	0.00	6.46	0.00
Soybean Oil	2.37	5.55	3.23	5.55
Lard	1.90	4.44	31.66	54.35

* Data were calculated based on the information provided by the vender.

**Table 2:** General metabolic characteristics.

	*BSA*	*Oleate*	*Palmitoleate*
Body weight (g)			
Prior to dosing	29.8±1.0	29.9±1.3	29.5±1.5
Post-dosing	30.4±1.5	29.9±1.3	29.4±1.5
Food intake (g/d/mouse)	2.68±0.0	2.73±0.1	2.86±0.1
Glucose (mg/dl)	216±15	204±11	170±11† *
Insulin (ng/ml)	1.6±0.3	1.5±0.2	1.0±0.3
Free fatty acids (mM)	0.4±0.1	0.4±0	0.5±0.1
Triglycerides (mg/dl)	37±1	37±0.2	37±1
HOMA-IR	10.8±1.7	10.2±1.2	5.7±1.7† *

Male wild-type C57BL/6J mice, at 5–6 weeks of age, were fed an LFD for 12 weeks and supplemented with palmitoleate (600 mg/kg/d, conjugated with bovine serum albumin (BSA) in phosphate-buffered saline (PBS)), oleate (600 mg/kg/d), or BSA (in PBS) via oral gavages for the last 4 weeks. After the feeding/supplementation regimen, mice were fasted for 4 hrs before collection of blood samples. Data are means ± SE, n = 4–6.

† *P*<0.05 palmitoleate vs. BSA;

* *P*<0.05 palmitoleate vs. oleate. HOMA-IR is an index of insulin resistance calculated according to the following equation. HOMA-IR  =  basal glucose (mmol/l) × basal insulin (mU/l)/22.5.

### Palmitoleate Supplementation Increases Fat Deposition, Improves Insulin Signaling, and Decreases the Inflammatory Response in Livers of LFD-fed Mice

**Figure 2:**
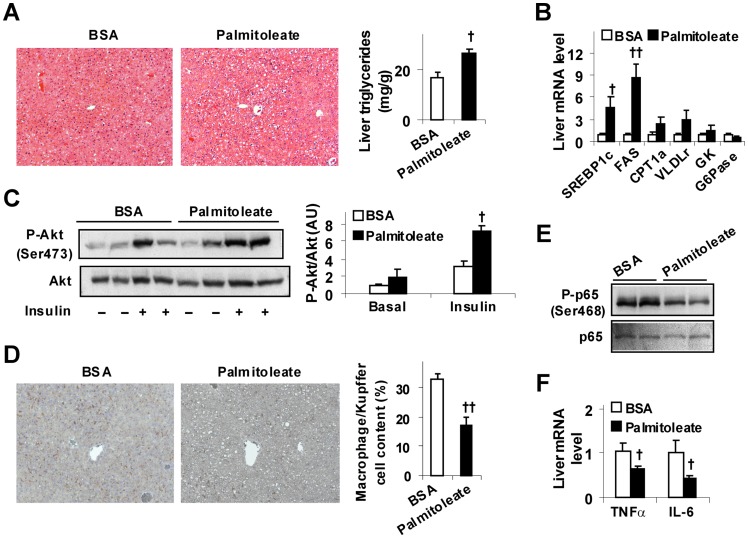
Induction of hepatic steatosis while improving liver insulin sensitivity and decreasing liver inflammatory response in LFD-fed mice. Male C57BL/6J mice, at 5–6 weeks of age, were fed a low-fat diet for 12 weeks and supplemented with palmitoleate (600 mg/kg/d), oleate (600 mg/kg/d), or bovine serum albumin (BSA) via oral gavages for the last 4 weeks. (A) Liver fat deposition. Right panels, liver sections were stained with H&E. Left panel, hepatic triglyceride levels. (B) Liver gene expression. SREBP1c, sterol-regulatory element-binding protein 1c; FAS, fatty acid synthase; CPT1a, carnitine palmitoyltransferase 1a; VLDLr, very low density lipoprotein receptor; GK, glucokinase; and G6Pase, glucose-6-phosphatase. (C) Liver insulin signaling. Livers of the treated mice were collected at 5 min after a bolus injection of insulin (1 U/kg) or PBS into the portal vein. Left panels, Akt and phospho-Akt (Ser473) were examined using Western blot analyses; right panel, quantification of P-Akt/Akt. AU, arbitrary unit. (D) Liver macrophages/Kupffer cells (left two panels, F4/80 staining; right panel, F4/80^+^ cell fraction). (E) Liver NF-κB p65 and phospho-p65 (Ser468). (F) Liver cytokine expression. For A – D and F, numeric data are means ± SE, n = 4–6. ^†^, *P*<0.05 and ^††^, *P*<0.01 palmitoleate vs. BSA (in A and D) for the same gene (in B and F) under the same condition (in C).

Given the positive correlation between the circulating levels of palmitoleate and hepatic steatosis in human subjects [16], the effect of palmitoleate on hepatic lipogenesis was determined. Because metabolic parameters of BSA-supplemented mice did not differ from those of oleate-supplemented mice, comparisons of the following data were made only between BSA-supplemented mice and palmitoleate-supplemented mice. Compared with controls, palmitoleate-supplemented mice exhibited a significant increase in liver fat deposition (Figure 2A). Additionally, palmitoleate-supplemented mice exhibited a marked increase in liver expression of genes for lipogenesis including sterol regulatory element-binding protein 1c (SREBP1c) and FAS, but not in liver expression of genes for fatty acid oxidation and very low density lipoprotein (VLDL) secretion such as CPT1a and VLDL receptor (VLDLr) (Figure 2B). When liver insulin signaling was analyzed, palmitoleate-supplemented mice exhibited a significant increase in insulin-stimulated Akt (Ser473) phosphorylation in livers (Figure 2C). Thus, palmitoleate induces hepatic steatosis while improving liver insulin sensitivity.

It remains largely unknown if palmitoleate alters liver inflammatory status, an essential factor that drives the progression of simple steatosis to steatohepatitis. Upon palmitoleate supplementation, the mice displayed a significant decrease in liver macrophage infiltration (17% vs. 33%, Figure 2D and Figure S2) compared with controls. Additionally, nuclear factor kappa B (NF-κB) p65 (Ser468) phosphorylation and tumor necrosis factor alpha (TNFα) and interleukin 6 (IL-6) expression in palmitoleate-supplemented mice were much lower than their respective levels in control mice (Figure 2E and F). Together, these results suggest that palmitoleate is an anti-inflammatory bioactive lipid that dissociates liver inflammatory response from hepatic steatosis; given the increase in liver fat deposition in palmitoleate-supplemented mice.

### Palmitoleate Dissociates Insulin Resistance and Liver Inflammatory Response from Hepatic Steatosis in HFD-fed Mice

**Figure 3:**
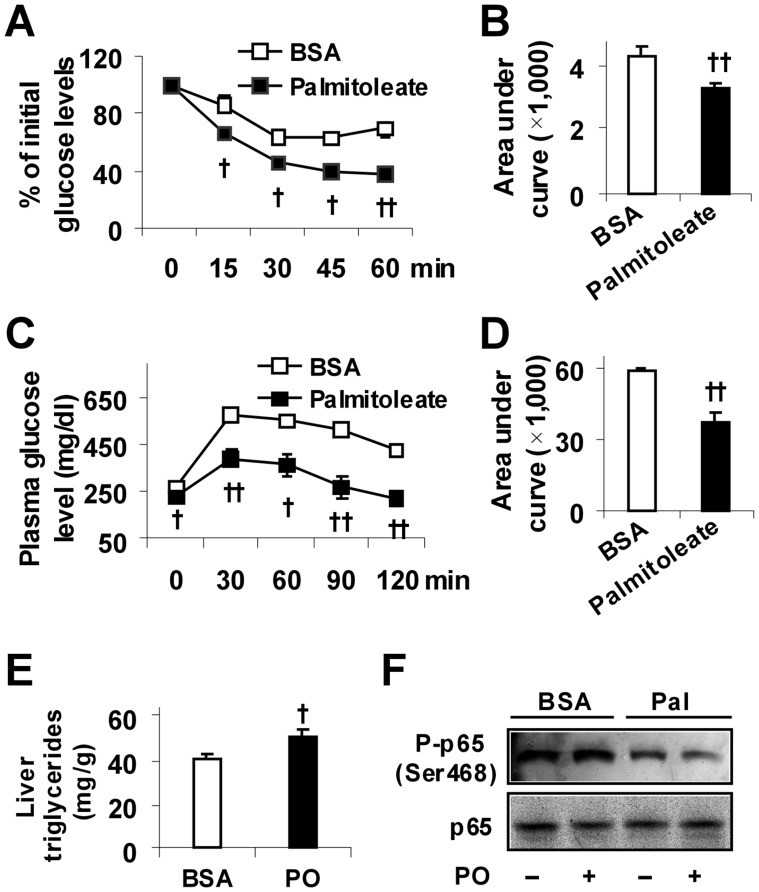
Improvement of systemic insulin sensitivity. Male C57BL/6J mice, at 5–6 weeks of age, were fed a high-fat diet for 12 weeks and supplemented with palmitoleate (PO) or bovine serum albumin (BSA) via oral gavages for the last 4 weeks. (A) Insulin tolerance tests (ITT). (B) Area under curve (AUC) was calculated based on ITT. (C) Glucose tolerance tests (GTT). (D) AUC was calculated based on GTT. For A and C, mice were fasted for 4 hrs and received an intraperitoneal injection of insulin (1 U/kg) (A) or glucose (2 g/kg) (C). (E) Hepatic levels of triglycerides. (F) Liver NF-κB p65 and phospho-p65 (Ser468). For A – E, data are means ± SE, n = 4–6. ^†^, *P*<0.05 and ^††^, *P*<0.01 palmitoleate vs. BSA (in B, D, and E) for the same time point (in A and C).

To confirm if palmitoleate brings about dissociation of insulin resistance, liver inflammatory response, and hepatic steatosis in mouse models of diet-induced obesity, wild-type mice were fed a high-fat diet (HFD, Table 1) and treated with or without palmitoleate. Compared with controls, palmitoleate supplementation improved systemic insulin sensitivity and glucose metabolic homeostasis in HFD-fed mice (Figure 3A–D). Additionally, in HFD-fed mice, palmitoleate supplementation caused a significant increase in hepatic levels of triglycerides and a significant decrease in the phosphorylation of NF-κB p65 (Ser468) in livers of the treated mice compared with controls (Figure 3 E and F). Thus, palmitoleate supplementation also dissociates insulin resistance and liver inflammatory response from hepatic steatosis in mice with diet-induced obesity.

### Palmitoleate Increases Fat Deposition, Stimulates FAS Expression, and Activates SREBP1c in Hepatocytes

**Figure 4:**
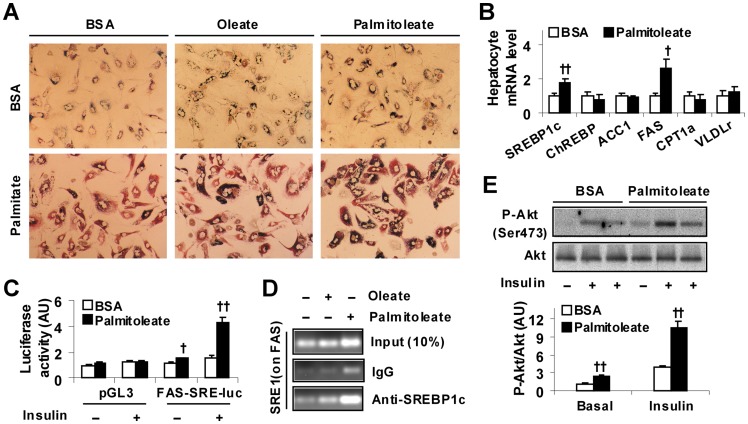
Induction of hepatocyte fat deposition while improving insulin signaling. (A) Hepatocyte fat deposition. Mouse primary hepatocytes were treated with palmitoleate (50 µM), oleate (200 µM), or BSA (in PBS) for 48 hrs in the presence or absence of palmitate (250 µM) for the last 24 hrs and stained with Oil-Red-O or 1 hr. (B) Hepatocyte gene expression. Mouse primary hepatocytes were treated with palmitoleate (50 µM) or BSA (in PBS) for 48 hrs. The expression of genes related to lipid metabolism was analyzed using real-time RT-PCR. ChREBP, carbohydrate responsive element-binding protein; ACC1, acetyl-CoA carboxylase 1. (C) Hepatocyte SREBP1c transcription activity. Mouse primary hepatocytes were transfected with a plasmid containing firefly luciferase reporter driven by SRE sequence on FAS gene (FAS-SRE-luc), or a control plasmid (pGL3-luc) for 24 hrs. After transfection, the cells were treated with palmitoleate (50 µM) or BSA for 48 hrs in the presence or absence of insulin (100 nM) for the last 24 hrs. (D) Hepatocyte ChIP assay. Mouse primary hepatocytes were treated with palmitoleate (50 µM), oleate (200 µM), or BSA for 48 hrs and subjected to the ChIP assay using antibodies against SREBP1c. The resultant DNA was analyzed by PCR with primers amplifying SRE-1 on FAS promoter. The input (control) contained 10% of each of the immunoprecipitants. (E) Hepatocyte insulin signaling. Cells were treated as described in (B). Prior to harvest, the cells were treated with or without insulin (100 nM) for 30 min. Top two panels, the levels and phosphorylation state of Akt (Ser473); bottom panel, quantification of P-Akt/Akt. AU, arbitrary unit. For B, C, and E, numeric data are means ± SE. All experiments were performed at least in quadruplicate. ^†^, *P*<0.05 and ^††^, *P*<0.01 palmitoleate vs. BSA for the same gene (B) under the same condition (C and E).

The direct effect of palmitoleate on fat deposition was examined in primary mouse hepatocytes. The dose of palmitoleate used was chosen based on published literature [20], [21]. As controls, the present study also examined lipid accumulation in primary mouse hepatocytes supplemented with oleate, whose dose was also chosen based on published literature [21], [22]. Compared with oleate and/or BSA, palmitoleate supplementation caused a slight but significant increase in lipid accumulation under the basal condition (BSA) (Figure 4A, top panels; quantitative data were shown in Figure S3). In the presence of palmitate, palmitoleate supplementation was still able to increase hepatocyte fat deposition (Figure 4A, bottom panels). When hepatocyte expression of genes related to major lipid metabolic pathways were analyzed, treatment with palmitoleate caused a significant increase in hepatocyte mRNA levels of SREBP1c and FAS, and showed no significant effect on hepatocyte mRNA levels of CPT1a and VLDLr (Figure 4B) compared with controls. These results suggest that increased lipogenesis appeared to account for an increase in hepatocyte fat deposition brought about by palmitoleate treatment. To address how palmitoleate increases hepatocyte FAS expression, the FAS reporter assay was performed. Compared with controls, treatment with palmitoleate increased SREBP1c transcription activity at the basal condition (without insulin) (Figure 4C). When insulin was supplemented, the effect of palmitoleate on increasing SREBP1c transcription activity was much more potent. Next, the chromatin immunoprecipitation (ChIP) assay was performed. Compared with BSA or oleate, palmitoleate treatment caused a marked increase in SREBP1c binding to FAS promoter (Figure 4D). The latter appeared to account for the effect of palmitoleate on increasing SREBP1c transcription activity. Although promoting hepatocyte fat deposition, palmitoleate increased hepatocyte Akt (Ser473) phosphorylation at both basal and insulin-stimulated conditions (Figure 4E), indicating improvement of hepatocyte insulin sensitivity.

### Palmitoleate Decreases the Inflammatory Response in Both Hepatocytes and Macrophages

**Figure 5:**
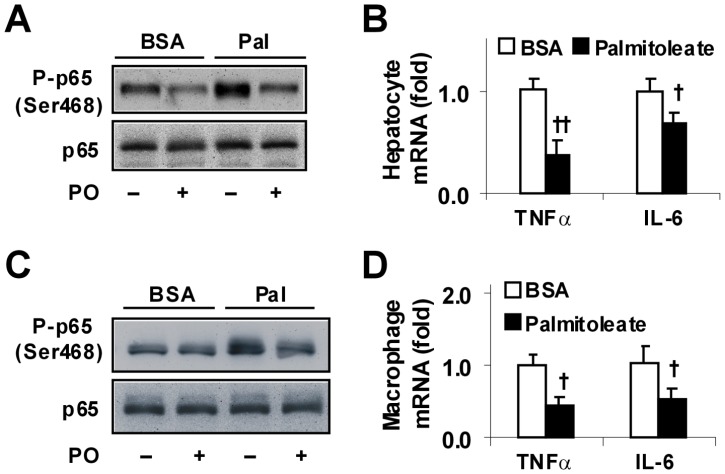
Suppression of inflammatory responses. Mouse primary hepatocytes and RAW macrophages were treated with palmitoleate (50 µM) or BSA (in PBS) for 48 hrs. (A) Hepatocyte NF-κB p65 and phospho-p65 (Ser468). (B) Hepatocyte expression of proinflammatory cytokines. (C) RAW macrophage NF-κB p65 and phospho-p65 (Ser468). (D) RAW macrophage expression of proinflammatory cytokines. For B and D, data are means ± SE. All experiments were performed in quadruplicate. ^†^, *P*<0.05 and ^††^, *P*<0.01 palmitoleate vs. BSA for the same gene.

The anti-inflammatory effect of palmitoleate was analyzed. Compared with controls, palmitoleate-supplemented hepatocytes exhibited a significant decrease in both basal and palmitate-stimulated NF-κB p65 (Ser468) phosphorylation and TNFα and IL-6 expression (Figure 5A and B). Additionally, treatment with palmitoleate caused a significant decrease in NF-κB p65 (Ser468) phosphorylation and TNFα and IL-6 expression in RAW macrophages in a pattern similar to that in mouse primary hepatocytes (Figure 5C and D). In combination with the above results on hepatocyte fat deposition (Figure 4), palmitoleate appears to dissociate hepatocyte inflammatory response from fat deposition.

## Discussion

The results from palmitoleate-supplemented mice support a causal role for palmitoleate in the development of hepatic steatosis. As substantial evidence, a direct effect of palmitoleate on inducing fat deposition was observed in mouse primary hepatocytes. Significantly, palmitoleate is pro-lipogenic, evidenced by increased hepatic expression of SREBP1c and FAS upon palmitoleate treatment. Furthermore, palmitoleate treatment activated SREBP1c transcription activity and increased the binding of SREBP1c to FAS promoter in hepatocytes. These aspects illustrate novel cellular mechanisms underlying how palmitoleate brings about fat deposition in liver/hepatocytes through stimulating lipogenesis. Initially, palmitoleate was thought to suppress liver lipogenesis [14]. This conclusion, however, appeared to be based only on a decrease in liver FAS expression in mice upon palmitoleate infusion during clamp. A similar conclusion was drawn in palmitoleate-supplemented KKAy mice [15], but also is inappropriate because decreased liver FAS expression occurred in a condition where palmitoleate-supplemented mice displayed a significant decrease in food intake. Given that food restriction has a powerful effect on reducing hepatic steatosis [23], the decrease in liver FAS expression in palmitoleate-supplemented KKAy mice could be attributed solely to reduction of food intake associated with palmitoleate dosing. In other words, the conclusion drawn from KKAy mice needs to be validated in mice upon pair-feeding. Because C57BL/6J mice were used in the present study, the possibility that mice with different genetic background respond differentially to palmitoleate can not be ruled out. Nonetheless, neither of the two previous mouse studies [14], [15] provided data to demonstrate a direct effect of palmitoleate on FAS expression in hepatocytes. In contrast, the present study provided both *in vivo* and *in vitro* results to argue in favor that palmitoleate stimulates hepatic events related to lipogenesis. As such, the present study offers a reasonable explanation for the positive correlation between the circulating levels of palmitoleate and hepatic steatosis observed in human subjects [16]. At this point, it is not sure why palmitoleate supplementation increased the circulating levels of oleate (18∶1n9) and linoelaidic acid (18∶2n6). It is also unknown if linoelaidic acid is pro-lipogenic.

Palmitoleate supplementation increased Akt (Ser473) phosphorylation in both livers and mouse primary hepatocytes. These effects further validate the insulin-sensitizing effect of palmitoleate [14]. Significantly, stimulation of Akt phosphorylation by palmitoleate was accompanied by increased fat deposition in livers/hepatocytes. The development of hepatic steatosis is thought to be largely due to insulin resistance [1], [24]. Thus, the present study indicates that palmitoleate dissociates hepatic steatosis from insulin resistance. Likely, palmitoleate acts through unrelated mechanisms to separately regulate lipogenesis and insulin signaling in hepatocytes. As mentioned above, palmitoleate directly increases hepatocyte SREBP1c activation, thereby bringing about increased FAS expression. Meanwhile, palmitoleate exhibits an anti-inflammatory property in hepatocytes (see below), which appears to account for the insulin-sensitizing effect of palmitoleate as the inflammatory status is a critical determinant of insulin sensitivity in hepatocytes [25], as well as other metabolic cells including adipocytes [26], [27]. When these two palmitoleate-driven events occur simultaneously, palmitoleate induces hepatic steatosis while increasing insulin signaling. This view is consistent with recent results from both human and mouse studies which increasingly indicate that fat deposition is not necessarily accompanied by insulin resistance [28], [29]. Indeed, in certain genetically modified mice, hepatic fat deposition is even inversely related to insulin resistance [30], [31], which is identical to the case in the present study. For this reason, hepatic steatosis may be a cost of improved insulin sensitivity at both systemic and hepatic levels. Thus, cautions should be taken when considering palmitoleate for insulin sensitization.

It is also a novel finding that palmitoleate supplementation suppressed liver inflammatory response. Notably, palmitoleate supplementation reduced the numbers of macrophages/Kupffer cells, and decreased the phosphorylation of NF-κB and the expression of proinflammatory cytokines in livers of the treated mice. In a previous study with human hepatoma cells, palmitoleate is shown to decrease palmitate-induced inflammatory signaling through the JNK pathway [19]. The later suggests a direct anti-inflammatory effect of palmitoleate. This is also the case in the present study. Upon palmitoleate supplementation, mouse primary hepatocytes exhibited a decrease in the phosphorylation of NF-κB and the expression of proinflammatory cytokines. Because of this, it is convincing that suppressing hepatocyte inflammatory response contributes to the *in vivo* actions of palmitoleate on liver inflammatory response. In the liver, macrophages/Kupffer cells critically determine liver inflammatory status [32]. Considering this, suppressing macrophage/Kupffer cell inflammatory response likely also contributes to the *in vivo* anti-inflammatory actions of palmitoleate. This view, indeed, is echoed by the fact that treatment with palmitoleate decreased the phosphorylation of NF-κB and the expression of proinflammatory cytokines in RAW macrophages. However, it is not clear about proportional contributions of hepatocytes and macrophages/Kupffer cells to palmitoleate actions on suppressing liver inflammatory response.

It should be noted that decreased liver/hepatocyte inflammatory response was accompanied by increased fat deposition in both livers and primary hepatocytes in response to palmitoleate supplementation. These effects were consistent with the results from Huh7 cells, in which palmitoleate increased steatosis but decreased the phosphorylation of JNK1/2 [19]. The underlying mechanisms by which palmitoleate dissociates the inflammatory response from steatosis remain to be elucidated, but could be attributable to the effect of palmitoleate on promoting esterification of free fatty acids [19]. Previously, a causal relationship between fat deposition and the inflammatory response has been established in hepatocytes [11], [12]. When the results from Huh7 cells [19] and from mouse primary hepatocytes of the present study were compared, the opposite consequences on the inflammatory response appear to be due to the differences in the types of fatty acids, i.e., palmitate vs. palmitoleate, from which triglycerides are synthesized. In other words, the type of fat deposited, but not the content fat is the key determinant of hepatocyte inflammatory response. The latter, as previously demonstrated, inversely correlates insulin sensitivity in adipocytes [26]. As additional evidence, a similar relationship between the inflammatory response and insulin sensitivity was observed in mouse primary hepatocytes. Collectively, it is obvious that the inflammatory response, but not fat deposition, inversely correlates with insulin sensitivity as discussed above.

In summary, the present study provides the first *in vivo* evidence to support a unique role for palmitoleate in dissociating liver inflammatory response from hepatic steatosis. Mechanistically, the pro-lipogenic effect of palmitoleate is manifested by increased hepatic FAS expression due to an increase in SREBP1c activation. Additionally, while promoting fat deposition, palmitoleate exhibits a direct anti-inflammatory effect on both hepatocytes and macrophages. Thus, palmitoleate supplementation could be useful for suppressing hepatic inflammatory response and for insulin-sensitization, but at a cost of inducing hepatic steatosis.

## Materials and Methods

### Animal Experiments

Wild-type C57BL/6J mice were maintained on a 12∶12-h light-dark cycle (lights on at 06∶00). At 5–6 weeks of age, male mice were fed an LFD (10% fat calories, detailed in Table 1) for 12 weeks and treated with palmitoleate (600 mg/d/kg body weight, conjugated with BSA and suspended in phosphate-buffered saline (PBS)), oleate (600 mg/d/kg body weight), or BSA via oral gavages for the last 4 weeks. During the 12-week feeding/treatment period, body weight and food intake of the mice were recorded weekly. After the feeding/treatment regimen, mice were fasted for 4 hrs before sacrifice for collection of blood and tissue samples [33]–[35]. Liver samples were either fixed and embedded for histological and immunohistochemical analyses or frozen in liquid nitrogen and then stored at –80°C for further analyses [25], [34]. Some mice were fasted similarly and used for insulin and glucose tolerance tests and insulin signaling analyses. For a separate study, male wild-type C57BL/6J mice, at 5–6 weeks of age, were fed an HFD (60% fat calories, detailed in Table 1) for 12 weeks and treated with palmitoleate or BSA via oral gavages for the last 4 weeks in the same way used for LFD-fed mice. The treated mice were then subjected to the assays to analyze hepatic steatosis and liver inflammatory response as described for LFD-fed mice. All animals received human care and all study protocols were approved by the Institutional Animal Care and Use Committee of Texas A&M University.

### Insulin and Glucose Tolerance Tests

Mice were fasted for 4 hrs and received an intraperitoneal injection of insulin (0.5 U/kg for LFD-fed mice and 1 U/kg for HFD-fed mice) or D-glucose (2°g/kg). For insulin tolerance tests, blood samples (5 µl) were collected from the tail vein before and at 15, 30, 45, and 60 min after the bolus insulin injection. Similarly, for glucose tolerance tests, blood samples were collected from the tail vein before and at 30, 60, 90 and 120 min after the glucose bolus injection [26], [36].

### Measurement of Metabolic Parameters

The levels of plasma glucose, triglycerides, and free fatty acids, as well as hepatic triglycerides were measured using metabolic assay kits (Sigma, St. Louis, MO and BioVision, Mountain View, CA). The levels of plasma insulin were measured using ELISA kits (Crystal Chem Inc., Downers Grove, IL). Additionally, plasma levels of glucose and insulin were used to calculate HOMA-IR, an indicator of systemic insulin resistance, using the following equation. HOMA-IR  =  basal glucose (mmol/l) × basal insulin (mU/l)/22.5.

### Lipid Profiling

Plasma and liver samples were subjected to lipid profiling. Total lipids were extracted using chloroform/methanol (2∶1, v/v) and separated by thin-layer chromatography. Different lipid fractions were analyzed by capillary gas chromatography [37].

### Histological and Immunohistochemical Analyses

The paraffin-embedded liver blocks were cut into sections of 5 µm thickness and stained with H&E. In addition, the sections were stained for the expression of F4/80 with rabbit anti-F4/80 (1∶100) (AbD Serotec, Raleigh, NC) as previously described [26]. The fraction of F4/80-expressing cells for each sample was calculated as the sum of the number of nuclei of F4/80-expressing cells divided by the total number of nuclei in sections of a sample. Six fields per slide were included, and a total of 4 to 6 mice per group were used.

### Cell Culture and Treatment

Mouse primary hepatocytes were isolated from C57BL/6J mice as previously described [34], [37]. To address direct effects of palmitoleate, the isolated hepatocytes were treated with palmitoleate (50 µM), oleate (200 µM), or BSA (in PBS) for 48 hrs in the presence or absence of palmitate (250 µM) for the last 24 hrs to induce fat deposition and the inflammatory response. To examine lipid accumulation, the treated hepatocytes were stained with Oil-Red-O. To determine changes in hepatocyte insulin signaling, the cells were treated with insulin (100 nM) or PBS for 30 min prior to harvest. Cell lysates were prepared and used to measure the levels of Akt1/2 and phospho-Akt (Ser473) using Western blots. Additionally, lysates of cells without insulin treatment were examined for the levels of NF-κB p65 and phospho-p65 (Ser468). To analyze hepatocyte gene expression, the total RNA was prepared and subjected to real-time RT-PCR. To gain insight into palmitoleate stimulation of FAS expression, some hepatocytes were used to examine the transcription activity of SREBP1c using the reporter gene analysis and the ChIP assay detailed below.

### Western Blots

Lysates were prepared from frozen tissue samples and cultured cells. Western blots were conducted as previously described [26], [36]. The levels of NF-κB p65, phospho-p65 (Pp65, Ser468), Akt1/2, and phospho-Akt (P-Akt, Ser473) were analyzed.

### RNA Isolation, Reverse Transcription, and Real-time PCR

The total RNA was isolated from frozen tissue samples and cultured hepatocytes. RNA isolation and real-time RT-PCR were conducted as previously described [35]. The mRNA levels were analyzed for SREBP1c, carbohydrate responsive element-binding protein (ChREBP), ACC1, FAS, CPT1a, VLDLr, glucokinase, glucose-6-phosphatase, TNFα, and/or IL-6.

### Gene Transcription Reporter Assay

Mouse primary hepatocytes were transfected with a plasmid containing firefly luciferase reporter driven by SRE sequence on FAS gene (FAS-SRE-luc), or a control plasmid (pGL3-luc) for 24 hrs as previously described [37]. After transfection, the cells were treated with palmitoleate (50 µM) or BSA for 48 hrs in the presence or absence of insulin (100 nM) for the last 24 hrs. Cell lysates were prepared and used to measure luciferase activity using a kit from Progema (Madison, WI).

### Chromatin Immunoprecipitation (ChIP) Assay

Mouse primary hepatocytes were treated with palmitoleate (50 µM), oleate (200 µM), or BSA for 48 hrs. Nuclear extracts were prepared and subjected to ChIP assay using the EZ-ChIP™ kit following the procedure provided (Millipore, Billerica, MA). Briefly, nuclear extracts were immunoprecipitated with rabbit anti-mouse SREBP1c antibody or donkey anti-rabbit IgG. The resultant DNA was analyzed by PCR with primers amplifying SRE-1 on FAS promoter as previously described [38].

### Statistical Methods

Numeric data are presented as means ± SE (standard error). Two-tailed ANOVA or Student’s *t* tests were used for statistical analyses. Differences were considered significant at the *P*<0.05.

## Supporting Information

Figure S1 **Liver lipid profile.** Male C57BL/6J mice, at 5–6 weeks of age, were fed an LFD for 12 weeks and supplemented with palmitoleate (PO), oleate (Ole), or bovine serum albumin (BSA) via oral gavages for the last 4 weeks. (A) Liver fatty acid profile in triglyceride fraction. (B) Liver fatty acid profile in fatty acid fraction. Data are means ± SE, n = 4–6.
(TIF)

Figure S2 **Staining of liver macrophages/Kupffer cells.** Male C57BL/6J mice, at 5–6 weeks of age, were fed an LFD for 12 weeks and supplemented with palmitoleate (PO), oleate (Ole), or bovine serum albumin (BSA) via oral gavages for the last 4 weeks. Liver sections were stained for macrophages/Kupffer cells (20×). Arrows indicate F4/80^+^ cells.
(TIF)

Figure S3 **Quantification of hepatocyte fat deposition.** Mouse primary hepatocytes were treated with palmitoleate (50 µM), oleate (200 µM), or BSA (in PBS) for 48 hrs in the presence or absence of palmitate (250 µM) for the last 24 hrs and stained with Oil-Red-O or 1 hr. Fat content was quantified using the colorimetric assay. AU, arbitrary unit. Data are means ± SE. All experiments were performed at least in quadruplicates. ^†^, *P*<0.05 palmitoleate vs. BSA or oleate under the same condition (BSA or palmitate).
(TIF)
