# Supplementary figures and images for: Palmitoleate Induces Hepatic Steatosis but Suppresses Liver Inflammatory Response in Mice
Source: PLoS One. 2012 Jun 29;7(6):e39286. doi: 10.1371/journal.pone.0039286 (PMC3387145; doi:10.1371/journal.pone.0039286)

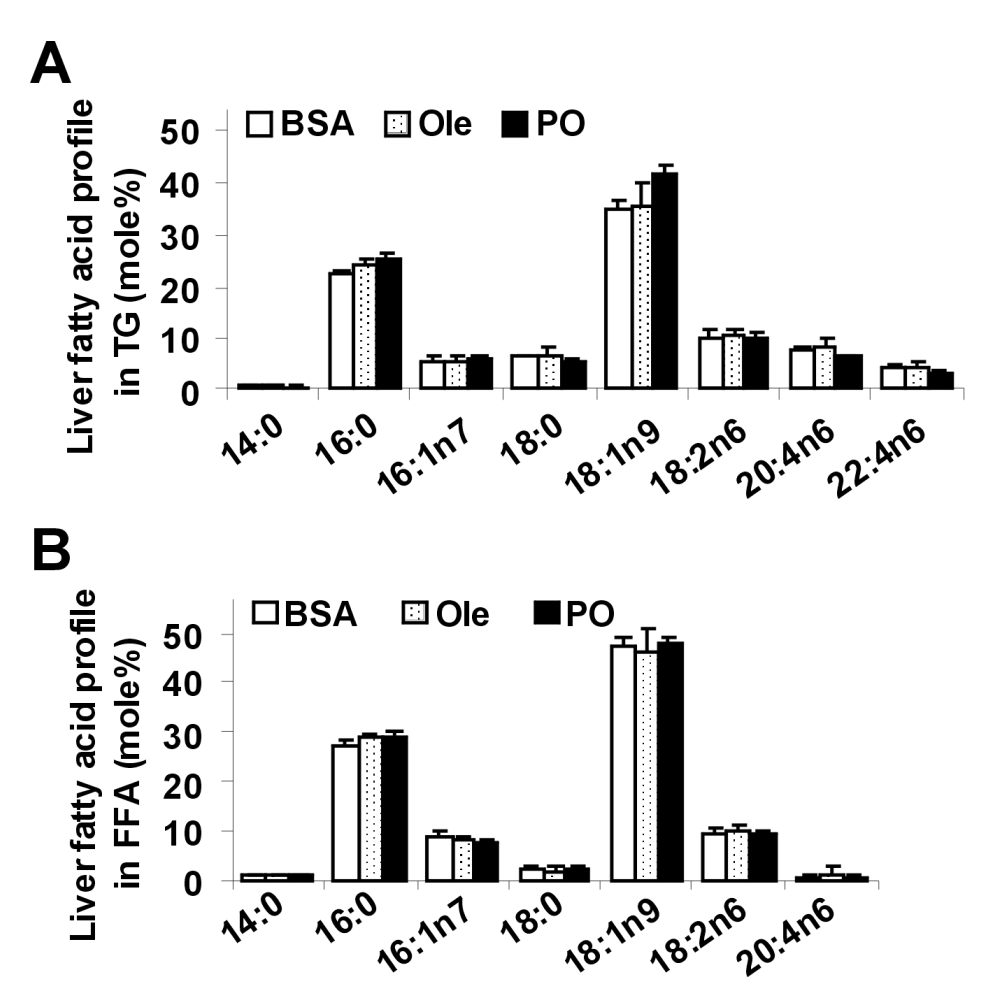

Supplement: Figure S1 — Liver lipid profile. Male C57BL/6J mice, at 5–6 weeks of age, were fed an LFD for 12 weeks and supplemented with palmitoleate (PO), oleate (Ole), or bovine serum albumin (BSA) via oral gavages for the last 4 weeks. (A) Liver fatty acid profile in triglyceride fraction. (B) Liver fatty acid profile in fatty acid fraction. Data are means ± SE, n = 4–6. (TIF) [file pone.0039286.s001.tif]

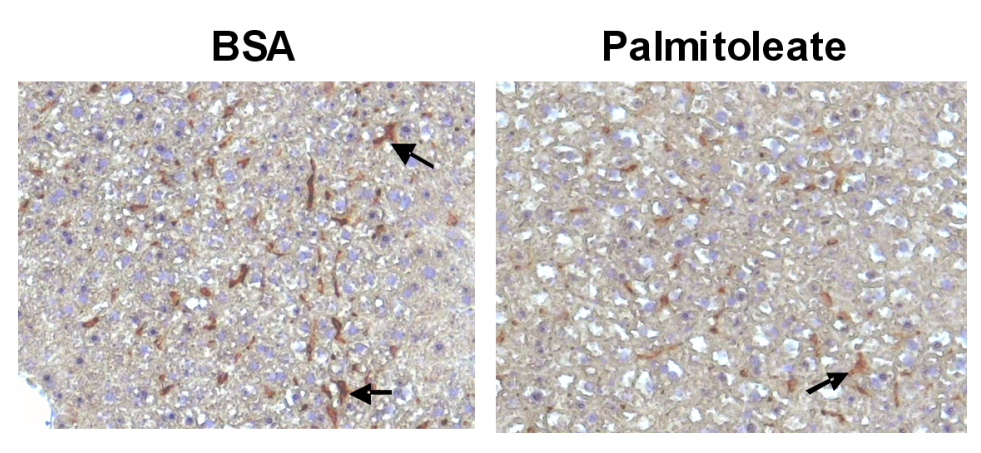

Supplement: Figure S2 — Staining of liver macrophages/Kupffer cells. Male C57BL/6J mice, at 5–6 weeks of age, were fed an LFD for 12 weeks and supplemented with palmitoleate (PO), oleate (Ole), or bovine serum albumin (BSA) via oral gavages for the last 4 weeks. Liver sections were stained for macrophages/Kupffer cells (20×). Arrows indicate F4/80+ cells. (TIF) [file pone.0039286.s002.tif]

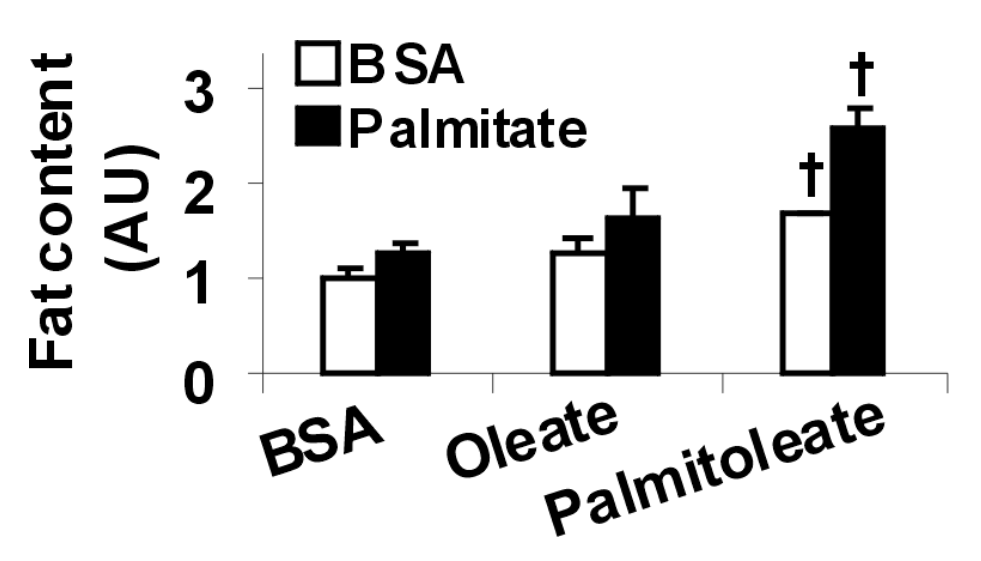

Supplement: Figure S3 — Quantification of hepatocyte fat deposition. Mouse primary hepatocytes were treated with palmitoleate (50 µM), oleate (200 µM), or BSA (in PBS) for 48 hrs in the presence or absence of palmitate (250 µM) for the last 24 hrs and stained with Oil-Red-O or 1 hr. Fat content was quantified using the colorimetric assay. AU, arbitrary unit. Data are means ± SE. All experiments were performed at least in quadruplicates. †, P<0.05 palmitoleate vs. BSA or oleate under the same condition (BSA or palmitate). (TIF) [file pone.0039286.s003.tif]
